# ^87^Sr/^86^Sr and ^14^C evidence for peccary (Tayassuidae) introduction challenges accepted historical interpretation of the 1657 Ligon map of Barbados

**DOI:** 10.1371/journal.pone.0216458

**Published:** 2019-05-14

**Authors:** Christina M. Giovas, George D. Kamenov, John Krigbaum

**Affiliations:** 1 Department of Archaeology, Simon Fraser University, Burnaby, British Columbia, Canada; 2 Department of Geological Sciences, University of Florida, Gainesville, Florida, United States of America; 3 Department of Anthropology, University of Florida, Gainesville, Florida, United States of America; The Cyprus Institute, CYPRUS

## Abstract

Contemporary West Indian biodiversity has been shaped by two millennia of non-native species introductions. Understanding the dynamics of this process and its legacy across extended temporal and spatial scales requires accurate knowledge of introduction timing and the species involved. Richard Ligon’s 17^th^ century account and celebrated map of early colonial Barbados records the translocation of several Old World species to the island in the post-contact era, including pigs (*Sus scrofa*) believed to have been released by passing sailors the century prior. Here we challenge this long-accepted historical narrative, presenting evidence that Ligon’s “pigs” were in fact peccaries, a New World continental mammal often confused with wild boars. We document the first recorded instance of non-native peccary (Tayassuidae) on Barbados based on a securely identified mandibular specimen from a historic archaeological context. Results of specimen ^87^Sr/^86^Sr and AMS radiocarbon assays, along with newly reported data from Sr isotope environmental analyses, indicate a local origin dating to AD 1645–1670/1780–1800. These data support the presence of living peccary on Barbados some time during the first 175 years of English settlement, which, based on review of historical and archaeological data, most likely arises from 16^th^ century peccary introduction from the Guianas/Trinidad by the Spanish or Portuguese. We argue dimorphic representations of “pigs” on Ligon’s map reflect the co-occurrence of peccary and European domestic swine on historic Barbados. Our findings overturn conventional history and provide greater taxonomic and chronological resolution for Caribbean bioinvasion studies, helping to refine our understanding of potential ecological impacts. In addition, the new bioavailable ^87^Sr/^86^Sr data for Barbados reported here advance current efforts toward mapping the Caribbean Sr isoscape.

## Introduction

Published in 1657, Richard Ligon’s volume *A True & Exact History of the Island of Barbados* has served as an important primary source for scholarly analysis of the early colonial West Indies and development of a plantation economy rooted in institutional slavery [1–3]. The volume is Ligon’s first-hand account of his 1647–1650 sojourn in the English colony of Barbados, founded in 1627. Set during the emergence of Barbados’ sugar industry, *A True & Exact History* provides insight into contemporary social and economic relations, the Atlantic slave trade, agro-industrial technology, and the political ramifications of the English Civil War (1642–1651). Ligon’s volume is also significant from a biogeographic perspective, documenting in rich narrative the island’s natural history and biodiversity. His species catalogue includes a detailed inventory of introduced Old World animals–primarily domesticated livestock–that presages the current crisis of biotic homogenization facing islands, while also highlighting the deeper antiquity of this phenomenon [4–7].

**Fig 1:**
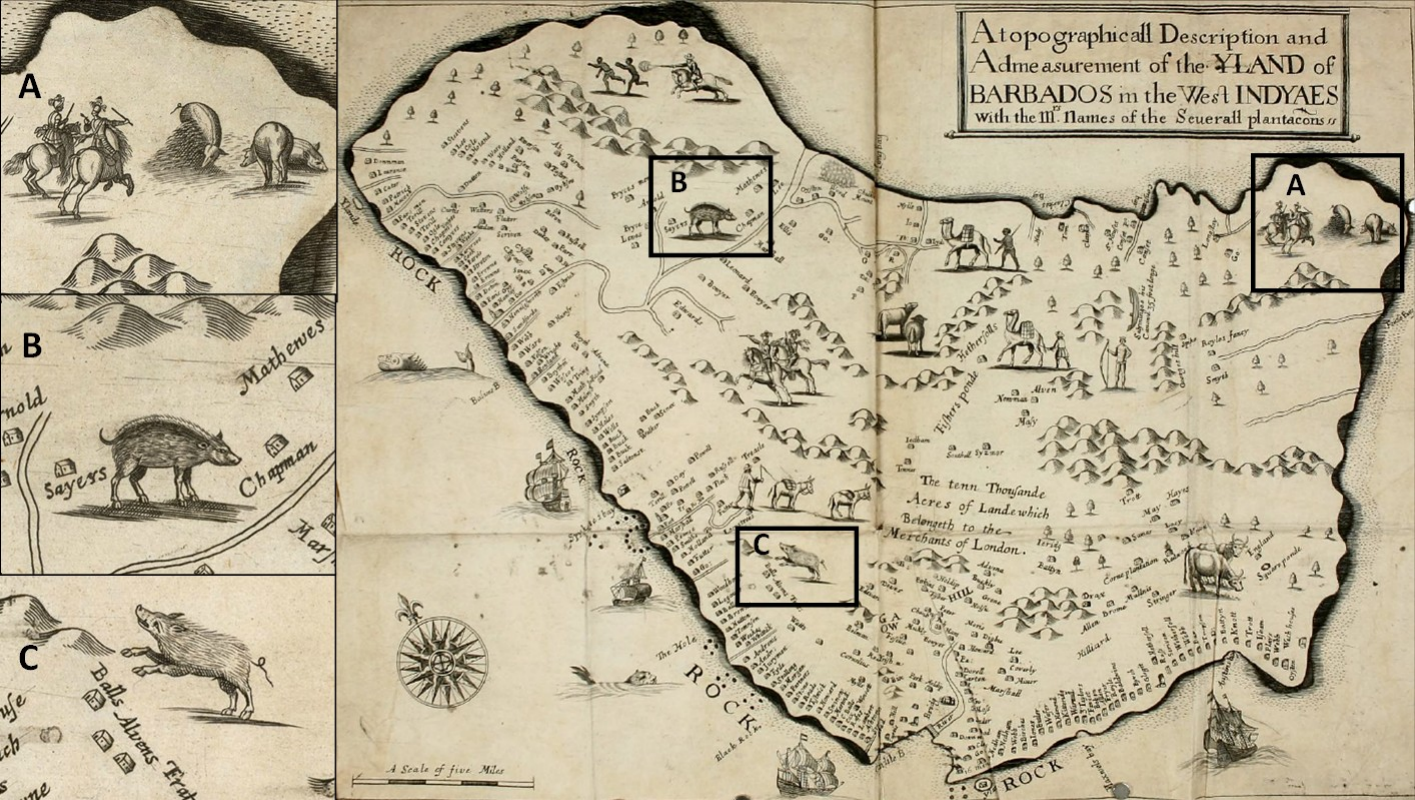
Map of Barbados published in *A True & Exact History of the Island of Barbados* (1657) by Richard Ligon. (A) smooth form “pig”; (B and C) hairy form “pig”. Barbados geographic coordinates: 13° 10′ 0″ N, 59° 32′ 0″ W. Map credit: public domain, https://www.biodiversitylibrary.org/item/113913#page/22/mode/1up CC BY-NC-SA 4.0 License; image cropped from original and adjusted for temperature and sharpness.

Barbados’ early colonial animal introductions are captured in the iconic map which accompanies Ligon’s volume (Fig 1), where sailing ships, a crowned Amerindian, and Europeans on horseback pursuing runaway slaves appear alongside donkeys, sheep, cattle, camels, and pigs (Fig 1A–1C). While elements of this map have been critiqued for their fanciful and imaginative quality [8], the presence of pigs seems unremarkable. During the 16^th^ and 17^th^ centuries it was common practice for sailing vessels to deposit domestic hogs (*Sus scrofa*) on islands to ensure a source of fresh meat upon future landings [9]. Ligon (p. 23 in [10]) explains their presence on Barbados as the result of translocation by visiting Portuguese sailors in the 16^th^ century, a time when the island was reportedly uninhabited. When the English arrived in 1625 they took note of the abundant hogs they encountered (p. 23 in [10]).

Ligon’s account of pig introduction has become conventional wisdom among scholars and is a fixture of Barbados’ colonial origin story [1, 3, 11, 12]. The pig constitutes a minor preoccupation for Ligon. He discusses the animal on ten occasions in *A True & Exact History*, with reference to the circumstances of their translocation, ostensible hunting by visiting Amerindians, husbandry, size, taste, and culinary preparation. Elements of this narrative, however, warrant greater critical scrutiny. The arrival of Old World fauna in the West Indies initiated dramatic changes in Antillean biodiversity and is responsible for significant population reductions and extinctions of endemic species through predation and competitive exclusion, in combination with effects from other human activities [13–16]. Understanding the mechanics and legacy of these processes requires knowledge of the timing of introductions and, in particular, a clear determination of the species involved, as their specific behaviours and life history traits interact dynamically with local conditions to determine ecological outcomes.

**Fig 2:**
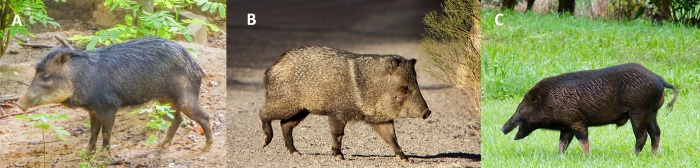
Physical similarities between peccaries and feral pig. (A) white-lipped peccary (*Tayassu pecari*); (B) collared peccary (*Pecari tajacu*); (C) feral pig (*Sus scrofa*). Photo credits: (A) -JvL-, *https://commons.wikimedia.org/wiki/File:White-lipped_peccary_at_the_Paramaribo_Zoo_(31332916405).jpg, CC BY 2.0 License, image cropped from original; (B) Alan Vernon, https://commons.wikimedia.org/wiki/File:Collared_Peccary_crossing_the_road.jpg, CC BY 2.0* License, image cropped from original; (C) Mike's Birds, *https://commons.wikimedia.org/wiki/File:Wild_pig_(37948082692).jpghttps://commons.wikimedia.org/wiki/File:Wild_pig_(37948082692).jpg, CC BY-SA 2.0 License, image cropped from original*.

At least one scholar has questioned Ligon’s identification of pig. In the mid-19^th^ century, Robert Schomburgk (p. 259 in [17]) speculated the animal in question was a species of peccary (Tayassuidae), mammals native to the continental Americas and Trinidad [18]. Peccaries strongly resemble feral pigs in their general form and hairiness (captive pigs possess little body hair) and were often confused with them up until the 19^th^ century (p. 3 in [9]) (Fig 2). Here, we report on the archaeological occurrence of a non-native peccary specimen from Barbados and present radiocarbon and strontium isotope evidence indicating a 17^th^-18^th^ century, local Barbados origin for this animal. Based on these data, we argue for a previously unrecorded introduction of peccary (*Tayassu pecari*/*Pecari tajacu*) to the island in the 16th century. We suggest the historical outcome of this event is reflected in Ligon’s 1657 map, which on close inspection reveals dimorphic illustrations of “pigs” that may represent both Old World swine and New World peccaries on the same landscape. This research contributes to ongoing efforts to clarify West Indian invasion biology and anthropogenic species translocations across deep time [13, 19–27]. Additionally, we provide new bioavailable ^87^Sr^/86^Sr data for Barbados that expand mapping of the West Indian Sr isoscape.

## Specimen archaeological background and identification

**Fig 3:**
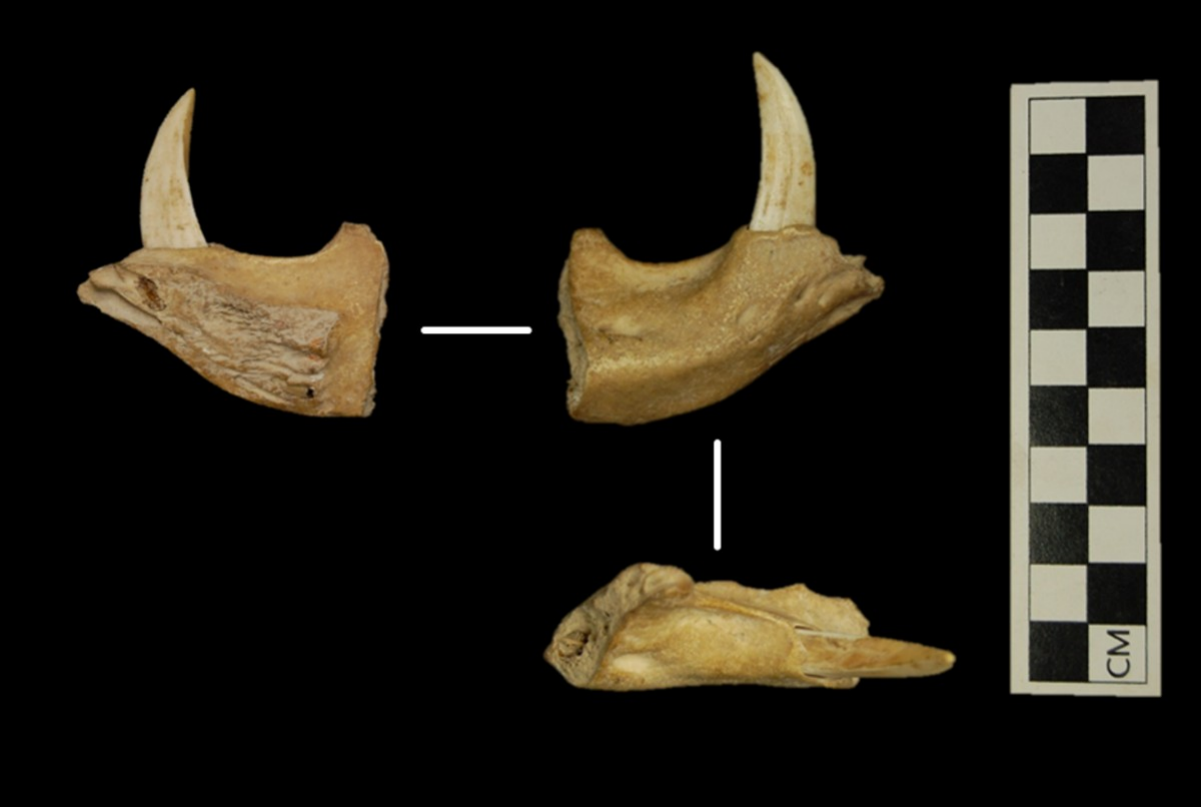
Right partial mandible of a peccary (Tayassuidae) collected from the Chancery Lane site, Barbados. From the collections of the Barbados Museum and Historical Society. Note the diastema lacking a premolar and the upward projecting canine, diagnostic attributes for peccary (Tayassuidae) that distinguish it from pig (*Sus scrofa*). Photos: CM Giovas.

**Fig 4:**
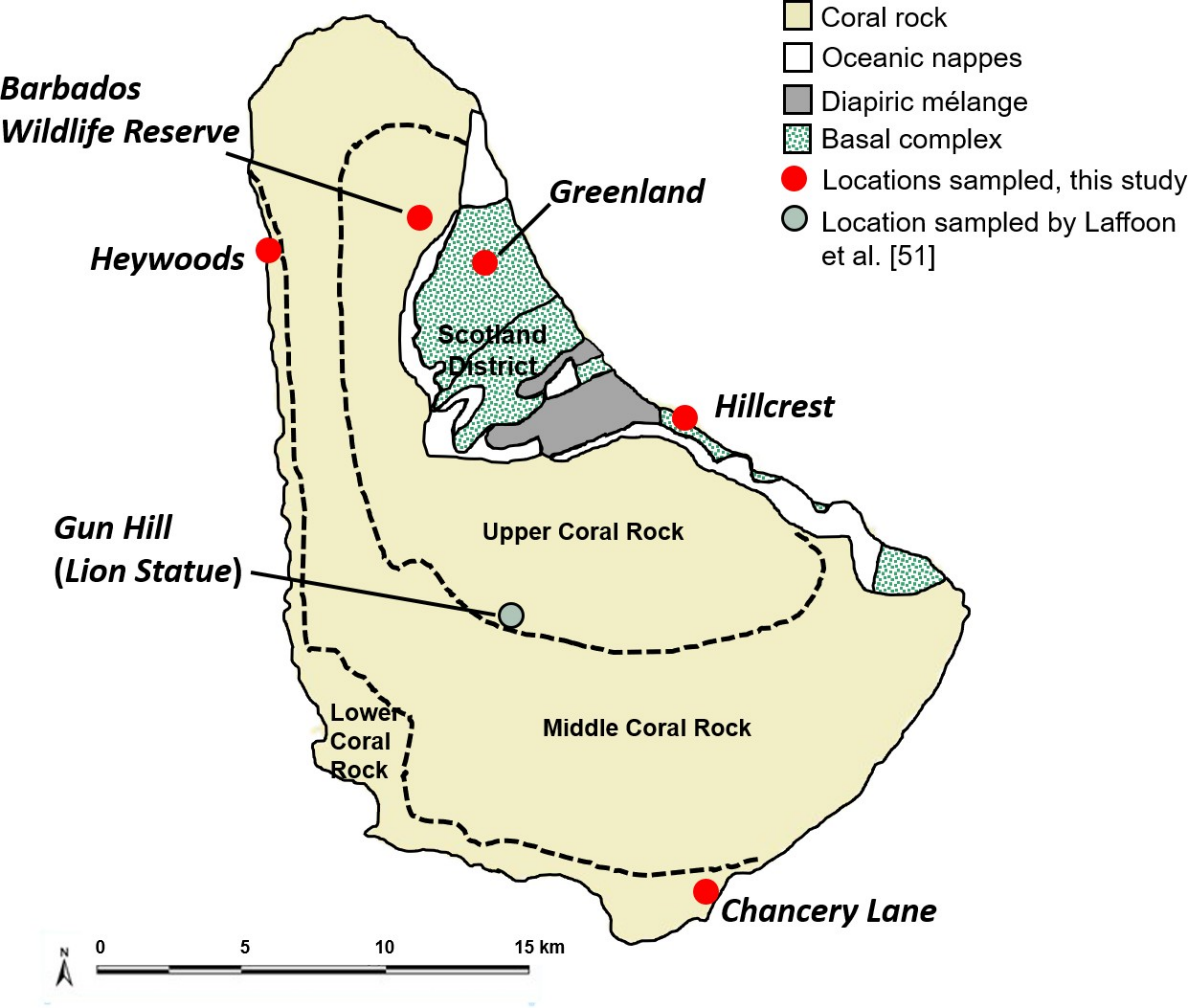
Geologic map of Barbados showing sample collection locations discussed in the text. (Adapted from Donovan and Harper [61]).

The specimen is a right, partial mandible, consisting of the anterior body, from the diastema to the mandibular symphysis (Fig 3). The adult canine is intact. Incisive alveoli are present, but the incisors are missing. The mandible was discovered in collections held by the Barbados Museum and Historical Society during a 2014 visit by CMG. The find lacked detailed contextual data, but associated information listed “Chancery Lane” as the collection site, accompanied by the name “Shilstone”, with “Connell” given as the “excavator” (Fig 4). Eustace Shilstone was the founder and first director of the Barbados Museum, and Neville Connell was his successor. Shilstone, Connell, and others conducted archaeological fieldwork at Chancery Lane in the 1930s to 1960s [28]. We believe it likely the peccary mandible was recovered in the course of this activity. The presence in the same find bag of a tooth from a historically introduced equid suggests the mandible specimen was collected from the ground surface or shallow historic deposits rather than the site’s prehistoric component (ca. AD 260–660).

The mandible is attributable to peccary (Tayassuidae) based on the cranially projecting canine, a diagnostic attribute that distinguishes it from pigs (*S*. *scrofa*), which possess a laterally projecting canine [29]. Species identification was not possible but is biogeographically constrained to either the white-lipped peccary (*T*. *pecari*), which ranges from Mexico to northern Argentina, or the collared peccary (*P*. *tajacu*), found in the southwest United States south to Argentina and on the island of Trinidad off the northeast coast of Venezuela [18, 30]. The rare Chacoan peccary is excluded from consideration here based on its limited distribution within a small area of Paraguay, Argentina, and Bolivia. Among peccaries, the adult canine typically erupts between 29–41 weeks [31] and grows until about 4 years of age [32]. The Barbados specimen thus represents an individual of at least 7 months, but likely older based on the degree of canine development. Unlike pigs, peccary canines do not grow continuously through life [29, 32].

Peccaries are not native to the biogeographic Caribbean; however, their skeletal remains have been found in prehistoric archaeological deposits on several Antillean islands [19, 33–37], indicating overwater transport by pre-Columbian Amerindians. White-lipped and collared peccaries were introduced to Cuba for game hunting in the 1930s [38]. To our knowledge, there are no historic or prehistoric records for peccary on Barbados [37, 38]. The unusual nature of the find and the specimen’s ambiguous archaeological context raised questions about how and when it reached Barbados. To clarify these issues, we conducted ^14^C dating and ^87^Sr^/86^Sr analysis on the specimen to determine its age and geographic origin.

## Materials and methods

### Sr isotopes in paleomobility studies

^87^Sr^/86^Sr ratios have been used extensively to detect ancient mobility in animals and humans, [24, 25, 39–43]. The means by which Sr inputs from the environment are incorporated biologically and the analytic considerations entailed in their measurement have been described in detail by others [39, 44–46] and are reviewed only briefly here. The ^87^Sr^/86^Sr analytic technique is based on the biological uptake of Sr isotopes from the environment through ingestion of food and water and their incorporation into skeletal tissues in equilibrium with local biologically available (bioavailable) levels. Owing to their large atomic mass, strontium isotopes do not fractionate substantively during biological processes. ^87^Sr^/86^Sr derived from tooth enamel thus corresponds roughly to averaged bioavailable Sr in the environment. Bioavailable Sr varies spatially based on geologic, atmospheric, and hydrospheric inputs. Non-local individuals are identified when their measured ^87^Sr^/86^Sr ratios depart significantly from that of the locality in which their skeletal remains were recovered, signaling an extra-local origin. Because tooth enamel apatite does not remodel and is less prone to diagenesis and contamination, it reflects local bioavailable ^87^Sr^/86^Sr at the time of mineralization and is preferred over bone for detecting human and animal mobility [44, 47, 48].

In the West Indies, ^87^Sr^/86^Sr analysis has been applied to understand patterns of human and animal movement between islands and the continent [22, 24, 25, 49–53] and reconstruct exchange networks through the sourcing of wood and animal-tooth artifacts [34, 35, 54]. Modelling and empirical studies conducted in the last decade have produced a more robust understanding of the ^87^Sr^/86^Sr isoscape of the islands and South American circum-Caribbean [22, 51, 54, 55], with recent efforts expanded to include multi-isotopic approaches [25, 34, 52] and evaluation of Sr inputs from African dust transport, notably from Saharan and Bodélé Depression sources, to the Caribbean [56, 57] (see also [58–60]).

Geologically, Barbados formed as an accretionary prism and is composed almost entirely of Tertiary and Quaternary marine strata [61–62] (Fig 4). Some 85% of the island consists of terraced Pleistocene reef limestones (coral rock). The remaining geology is composed of older sedimentary rocks, which occupy a northeastern section of Barbados, extending southeast along the coast. Known as the Scotland District, this area comprises Eocene to Miocene aged oceanic nappes that incorporate debris from the Antillean volcanic arc; a diapiric mélange (mudstone, sand, clay, ironstone, calcitized radstone, quartz sandstone, and chert); and a basal complex formed from accretion during the late Eocene of sediment (siliciclastic sedimentary rocks of turbiditic origin and hemipelagic, radiolarian-rich clays) scraped from the Lesser Antilles Accretionary Prism [61].

Published data on bioavailable ^87^Sr^/86^Sr for Barbados is limited to a single snail sample collected from a Pleistocene reef terrace [51] (Fig 4), with a Sr ratio (0.70918, ± 2 S.E. = 0.00001) consistent with the expected influence of seawater Sr in Barbados’ reef limestone. While Barbados’ marine strata should reflect Cenozoic Sr ratios of seawater, which ranged from 0.7078 to 0.7092 [63], bioavailable Sr may depart from these values based on the nature of bedrock weathering and other inputs. Applying a multi-source model incorporating sea salt, mineral dust aerosols, and bedrock weathering, Bataille et al. [53] predicted bioavailable Sr ranges of 0.708–0.709 for the Scotland District and 0.709–0.710 for the remainder of Barbados. Kumar et al. [56] recently confirmed long-range, seasonally variable transport to Barbados of Saharan dust with ^87^Sr^/86^Sr ratios lying between 0.718–0.723. However, dust samples in that study were filter-captured from an elevated tower. The degree to which exogenous aerosols influence bioavailable Sr for Barbados thus requires further empirical assessment. Notably, Schulting et al. [57] demonstrated an insignificant effect for Saharan dust on bioavailable Sr in the Bahamas, contrary to Bataille et al.’s [55] model predictions.

### Sample preparation and analysis

A 1 g piece of bone was cut from the peccary mandible using an NSK dental drill and a diamond grit rotary wheel and sent to the Keck Carbon cycle AMS Facility at the University of California, Irvine for accelerator mass spectrometry (AMS) assay. Bone collagen was extracted and purified using a modified Longin method with ultrafiltration following methods described by Kennett et al. [64]. The sample bone was cleaned of surficial sediment and the exposed surfaces were removed with a scalpel. The sample was demineralized for 24–36 h in 0.5N HCl at 5°C followed by a short (<1 h) alkali bath in 0.1N NaOH at room temperature to remove humates. The residue was then rinsed to neutrality in multiple changes of 18.2MΩ/cm H_2_O and gelatinized for 12 h at 60°C in 0.01N HCl. The resulting gelatin was lyophilized and weighed to calculate percent yield and assess collagen preservation. Rehydrated gelatin solution was pipetted into pre-cleaned Centriprep [65] ultrafilters (retaining >30 kDa molecular weight gelatin). The solution was centrifuged three times for 20 min, diluted with 18.2MΩ/cm H_2_O, then centrifuged three more times for an additional 20 min to desalt the solution.

The purified samples was combusted at 800°C in vacuum-sealed quartz tubes with CuO and Ag wires for 3 h. Sample CO_2_ was reduced to graphite at 550°C using H_2_ and a Fe catalyst, with reaction water drawn off with Mg(ClO_4_)_2_ [66]. Graphite samples were pressed into targets in Al cathodes and loaded on the target wheel for measurement on an NEC 1.5SDH 500kV compact accelerator mass spectrometer (AMS). The ^14^C age was corrected for mass-dependent fractionation with the measured δ^13^C value [67] and compared with samples of Pleistocene whale bone (backgrounds, 48,000 ^14^C BP), late Holocene bison bone (~1,850 ^14^C BP), and OX-2 oxalic acid standards for calibration. Splits of the processed ultrafiltered gelatin were measured for carbon and nitrogen concentrations and stable isotope ratios at the Yale Analytical and Stable Isotope Center with a Costech elemental analyzer (ECS 4010) and Thermo DeltaPlus analyzer. Sample quality was evaluated by % crude gelatin yield, %C, %N and C:N ratios before AMS ^14^C dating. Radiocarbon calibration was performed with Calib 7.1 based on the IntCal 13 calibration curve [68]. Calibrated dates reported in this study are rounded outward to the nearest five years.

For ^87^Sr^/86^Sr analysis, a section of enamel from the canine was sampled and prepared in the University of Florida Department of Anthropology’s Bone Chemistry Lab and the clean lab facilities of the Department of Geological Sciences following protocols described in Giovas et al. [22] and Valentine et al. [69]. In addition to the peccary canine, we analyzed 12 environmental samples collected from locations around the island to characterize bioavailable Sr in Barbados (Fig 4; Table 1). These comprise geologic samples (carbonate rock, n = 2; soil, n = 3), modern plant material (n = 2), modern (n = 1) and archaeological (n = 3) terrestrial snail shell, and tooth enamel from a prehistoric dog burial from the Heywoods archaeological site (n = 1).

**Table 1:** ^87^Sr^/86^Sr ratios of rock, soil, plant, and animal samples from Barbados.

Isotope Lab #	Location/Site	Sample Type	Species/ID	^87^Sr/^86^Sr	± S.E
L	Chancery Lane	C_1_ tooth enamel	*Pecari/Tayassu*	0.70898	0.000013
BBD-CL-2	Chancery Lane	soil acetic leach		0.70914	0.000016
SH-14-13	Chancery Lane	archaeological terr. snail shell	undetermined	0.70907	0.000012
SH-14-12	Greenland	archaeological terr. snail shell	undetermined	0.70808	0.000020
SH-14-18 (BBD-8)	Barbados Wildlife Reserve	modern terr. snail shell	undetermined	0.70918	0.000018
BBD-7	Barbados Wildlife Reserve	carbonate rock		0.70911	0.000010
BBD-9	Barbados Wildlife Reserve	modern plant	undetermined shrub	0.70916	0.000014
BBD12	Hillcrest	carbonate rock		0.70915	0.000008
BBD-11	Hillcrest	modern plant	*Canavalia rosea*	0.70874	0.000013
SH-14-11	Heywoods	archaeological terr. snail shell	undetermined	0.70918	0.000011
BBD-HY-1	Heywoods	archaeological dog burial, C_1_ enamel	*Canis lupus familiaris*	0.70912	0.000015
BBD-HY-2	Heywoods	soil acetic leach		0.70918	0.000015
BBD-HY-3	Heywoods	soil acetic leach		0.70919	0.000010
Laffoon et al. [51]	Lion Statue (Gun Hill)	modern terr. snail shell	unreported	0.70918	2± S.E 0.00001
			Mean (excluding peccary):	0.709036	
			σ:	0.000299	

Tooth samples were abraded using an NSK dental drill fitted with a 4 mm diamond tipped Brasseler bit to remove surficial contaminants. For the peccary canine, an enamel segment was then cut away using an NSK dental drill and a Dedeco separating disc. Dentine and cementum were removed from peccary and dog enamel samples using a Brasseler tapered diamond bit and binocular microscope. Enamel fragments were then powdered using an agate mortar and pestle, and ~25 mg of sample powder was placed in a microcentrifuge tube and treated with a 2% bleach (NaOHCl) solution for 8 h at room temperature to remove organic contaminants. Samples were rinsed to neutral pH with 2X H_2_0, then treated with 0.2 M acetic acid (CH_3_COOH) for an additional 8 h at room temperature, rinsed once again to neutral, frozen, and lyophilized.

In the clean lab, Versa Clean was used to preclean Teflon vials, which were then bathed in 8N HNO_3_ for 24 h, followed by 6N HCl for 24 h and repeated rinsing in MilliQ H_2_O. 3 ml 6N HCl was added to each individual vial, capped, and refluxed overnight at 120°C on a hot plate. Following the reflux, the HCl was discarded and each vial was rinsed with quadruple de-ionized water (4X H_2_O) and dried under a laminar flow hood. Pretreated enamel powder was then weighed into pre-cleaned Teflon vials and dissolved with 3 ml 50% nitric acid (HNO_3_) and evaporated to dryness on a hot plate at 120°C under a laminar flow hood.

To prepare terrestrial snail shells for Sr analysis, loosely adhering organics and sediment were first removed from the shell with a scalpel before cutting away a ~1 cm^2^ segment with a Dremel tool fitted with a diamond cutting wheel. Shell samples were then sonicated in distilled water for 10 min to remove any remaining surface contaminants. Once dried, samples were weighed and placed in pre-cleaned Teflon vials and pretreated with 2 ml of 30% hydrogen peroxide (H_2_O_2_) to remove organics, rinsed to neutral pH, and then 0.5 ml 5% acetic acid (CH_3_COOH) was added. Acetic acid was pipetted off and samples flushed with 1 ml 4X H_2_O, which was extracted after ~5 min. A further 1 ml 4X H_2_O was added to sample vials, followed by 0.5 ml 50% nitric acid (HNO_3_) to dissolve the shell. Sample vials were then placed uncapped on a hot plate at 120°C and evaporated to dryness under a laminar flow hood.

Geologic materials analyzed consisted of carbonate rocks detached directly from outcrops and sediment samples collected from loose material housed within the terrestrial snail shells. Rocks were broken apart to obtain fragments with entirely clean surfaces, which were then powdered by hand with a pestle and mortar. Powdered rock samples (~50 mg) were loaded into pre-cleaned Teflon vials and dissolved in 1 ml 50% nitric acid (HNO_3_). Vials were placed uncapped on a hot plate at 120°C and evaporated to dryness under a laminar flow hood. Soil samples were prepared for analysis following sequential leaching protocols in Kamenov [70], with resultant acetic leachates capturing the bioavailable component of soil Sr.

Plant samples (~1 g) were placed in clean ceramic crucibles and ashed at 550°C for 6 h. The ash was transferred to clean vials and leached with 5 ml 2N HCl for 24 h. The HCl leachate was subsequently transferred to precleaned Teflon vials and evaporated to dryness following protocols in Valentine et al. [69].

Ion chromatography was used to separate Sr from aliquots using procedures described in Valentine et al. [69]. Samples were dissolved in 3.5 N HNO_3_ and loaded onto cation exchange columns packed with Sr-selective crown ether resin (Sr spec, Eichrom Technologies, Inc.) to separate Sr from other ions, based on protocols outlined by Pin and Bassin [71].

^87^Sr/^86^Sr ratios were measured via a NuPlasma multi-collector inductively-coupled plasma-mass spectrometer (MC-ICP-MS) in the Department of Geological Sciences at the University of Florida using time-resolved analysis (TRA). ^87^Sr/^86^Sr ratios are reported relative to NBS 987 = 0.710246 (±0.00003, 2σ). Determination of the Sr concentration in each sample was made semi-quantitatively by comparing the signal intensity (V) of ^88^Sr during analysis with the ^88^Sr signals of NBS 987 standards with known Sr concentrations. Measured procedural blanks were 100 pg.

## Results

### AMS dating

AMS dating yielded an age of 235 ± 15 BP (UCIAMS-181681; δ^13^C: -21.5 ‰; δ^15^N: 8.0 ‰; 45.4%C; 14.8%N), with a 2σ calibrated range of AD 1645–1670/1780–1800 and median probability of AD 1660 [68]. The C:N ratio (3.58), a proxy measure for collagen quality, falls within the acceptable range of 2.9–3.6 [72, 73], with the ultrafiltered gelatin reported as white, high quality, and showing no signs of contamination from humates or conservants (B. Culleton, personal communication 2018). The two calibrated date ranges, representing 69.7% and 30.3% probability distributions respectively, arise from multiple intercepts of the calibration curve for the measured age. These dates place the peccary specimen firmly within the first 175 years of English settlement on Barbados and possibly coeval with the timing of Ligon’s visit to Barbados.

### ^87^Sr/^86^Sr analysis

**Fig 5:**
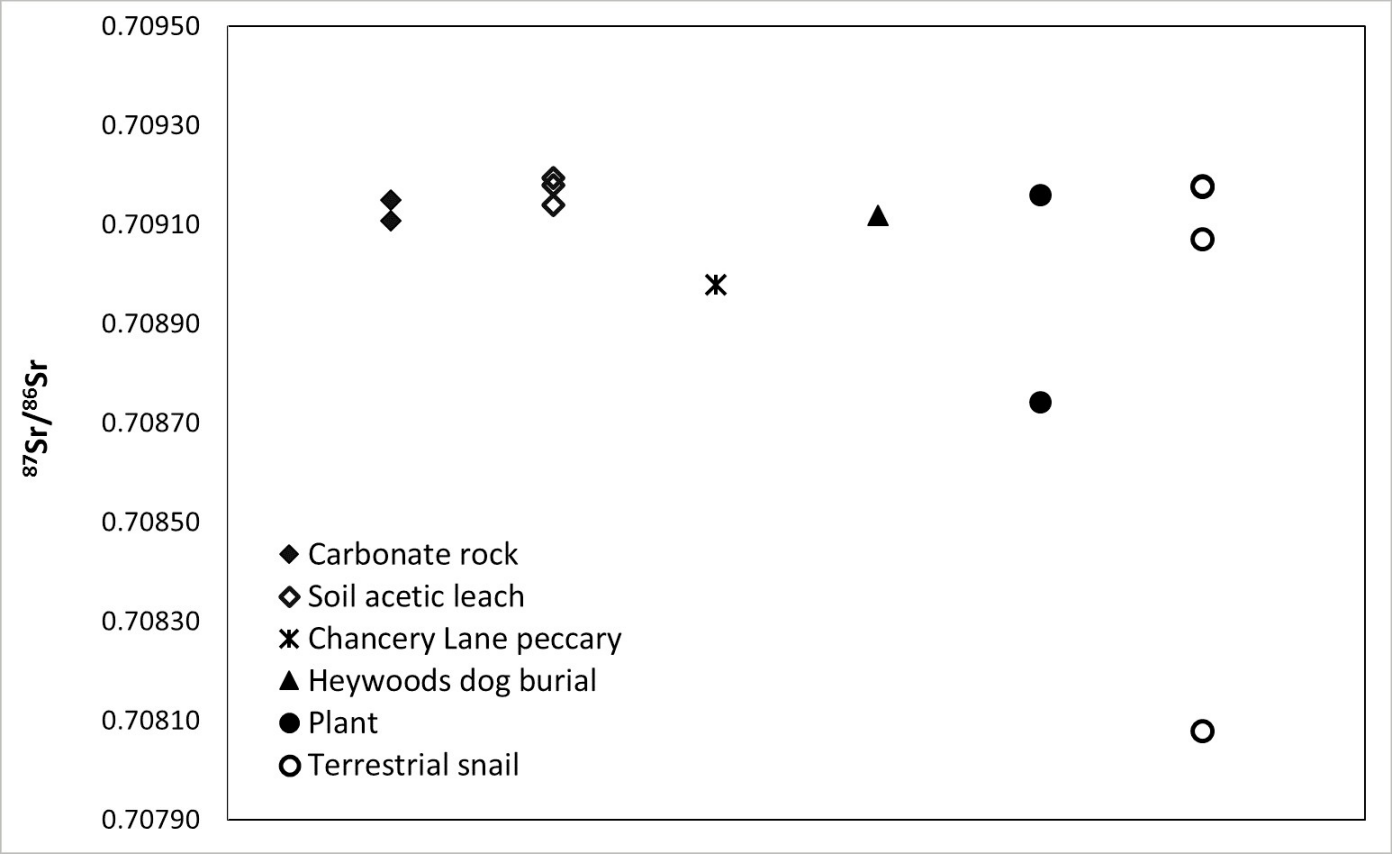
Plotted results of ^87^Sr/^86^Sr analysis.

^87^Sr/^86^Sr results (Table 1, Fig 5) range from 0.70808 to 0.70919 and are consistent overall with expectations based on Barbados’ marine carbonate geology as well as the single Sr analysis of 0.70918 previously obtained by Laffoon et al. [51] on modern snail (Lion Statue/Gun Hill sample, Table 1). The archaeological land snail (SH-14-12) from the Greenland site, within the Scotland District, plots low (0.70808) but is consistent with modeled Sr ratios of 0.707–0.708 for this geologically distinct area [55]. The prehistoric dog (BBD-HY-1) from the Heywoods site exhibits a local signal (0.70912), which contrasts with findings for the interisland transport of dogs elsewhere in the pre-Columbian Antilles [24, 25]. Our Sr results show a tight correspondence between bioavailable Sr and local geology and do not support a dust signal from Saharan or Bodélé Depression sources in biologically available Sr, although additional study is needed.

Excluding the peccary from calculation results in a mean bioavailable ^87^Sr/^86^Sr of 0.709036 (σ = 0.000299, n = 13, Lion Statue/Gun Hill specimen included). The ^87^Sr/^86^Sr for the Chancery Lane peccary specimen (BBD-CL-1; 0.70898) accords with this local signal. The surrounding Lesser Antilles exhibit significantly lower mean ^87^Sr/^86^Sr ratios (< 0.7083) than Barbados, while northern South America exhibit significantly higher mean ratios (> 0.7095) [22, 51].

The closest island with a similar mean Sr ratio to Barbados is Marie Galante ($\bar{x}=0.70917$, n = 4) [51], a carbonate island 330 km to the northwest, where peccary is unknown historically and prehistorically. While Sr ratios for Trinidad generally fall above ~0.7100, localized areas in southern Trinidad exhibit bioavailable Sr comparable to Barbados (~0.7090) (Fig 11 in [54]). *P*. *tajacu* occurs naturally on Trinidad [18], which was connected to mainland South America until the end of the Pleistocene. It is possible the Chancery Lane mandible originated in southern Trinidad (or another discrete area isotopically similar to Barbados) and was imported subsequently to Barbados as a live animal or carcass. However, peccaries have relatively large home ranges, up to 8 km^2^ for *P*. *tajacu* [74] and up to 200 km^2^ for *T*. *pecari* [75], depending on herd size and environment. Their isotopic ratios are unlikely to reflect localized areas, but rather mixing of bioavailable Sr contributions across a wider region. In a recent study by Krigbaum et al. [76], archaeological human and faunal remains from Trinidad showed ^87^Sr/^86^Sr from 0.7095 to 0.7134, which falls well above the Barbados samples analyzed in this work. We anticipate that a Trinidadian peccary would incorporate bioavailable Sr from multiple isotopic provinces within the island and depart from the values around ~0.7090 that characterize some areas of southern Trinidad. Indeed, a prehistoric collared peccary sample from the Red House site exhibited a ^87^Sr/^86^Sr ratio of 0.71032, significantly higher than the Barbados peccary [76]. Given this, we consider it most likely that that the Chancery Lane peccary specimen represents an animal that was living on Barbados sometime between 1645 and 1800.

## Discussion

This study provides evidence for a previously unrecognized peccary introduction to Barbados and indicates the presence of this animal on the island in the 17^th^ or 18^th^ century. Along with this new record of human-assisted introduction, our results expand bioavailable Sr data for Barbados and contribute to ongoing efforts to map the West Indian Sr isoscape [22, 25, 34, 51, 54, 55, 57]. Further investigation is required to determine the species, timing, source, and number of animals involved in peccary translocation, and the size and persistence of the population on the island.

Below, we consider three primary explanations for the translocation of peccary to Barbados, the relative likelihood of each based on available historical and archaeological data, and the implications for Richard Ligon’s treatise. In light of the low probability of recovering skeletal material from the failed introduction of a single animal, we make the working assumption that the Chancery Lane peccary was one among multiple individuals on Barbados at this time. While it is possible that the animal was introduced to the island at an early age, with canine development occurring after blood-level Sr had equilibrated with local environmental Sr, our discussion below incorporates the potential for this peccary to have been born from pre-existing peccary herds on Barbados. These assumptions carry ecological significance as they bear on the magnitude and duration of non-native species impact.

### Explanation 1: A pre-Columbian Amerindian introduction

Ligon (pp. 23–24 in [10]) reports disputed settler stories of Amerindians from the Lesser Antilles visiting Barbados to hunt pigs. Regardless of whether such visits occurred, an indigenous connection to pig/peccary is plausible. The pigs encountered by the English could have in fact been peccaries left by the pre-contact Amerindians of Barbados, who settled the island by 2430–1990 BC [77, 78]. Animal translocation becomes a widespread occurrence in the prehistoric West Indies after the first century AD [19, 20, 37]. Non-native peccary skeletal remains are recorded from pre-Columbian archaeological sites on at least half a dozen islands, including nearby Carriacou [21], Grenada [79], and possibly St. Vincent [80], although some of these specimens are modified for ornamental use and may represent trade items. No such finds are recorded for Barbados, however, where dog is the only known prehistorically introduced mammal [37]. If the animals noted by the early colonial English were indeed descendants of pre-Columbian peccaries, then this negative archaeological evidence suggests those populations were either small or rarely exploited by Amerindians. Based on available evidence, a pre-Columbian origin for peccary introduction appears unlikely.

### Explanation 2: A Spanish or Portuguese introduction

During the Age of Exploration, it was common for passing sailors to release a breeding pair of pigs or goats on an island to ensure a source of fresh meat upon future landings [19]. Conventional accounts [1, 3, 11, 12] hold that this occurred on Barbados in the 16^th^ century. As noted earlier, Ligon (p. 23 in [10]) reports settler stories of an island almost overrun by pigs when the English arrived and attributes this introduction to the Portuguese. Others credit the Spanish (p. 20 in [1]). Both had reached Barbados by the first half of the 16^th^ century, although neither established settlements. The Spanish and Portuguese, as well as Dutch and English, had a colonial presence in Trinidad and along the eastern coast of South America by the mid to late 16^th^ century. Columbus visited Trinidad on his third voyage in 1498, and the city of Port of Spain was founded in 1560. The Portuguese began colonizing Brazil in 1532, and the English, under the command of Walter Raleigh explored the region in the 1590s. It may be that one of these parties introduced not pig but peccary from the continent to Barbados on a passing voyage.

**Fig 6:**
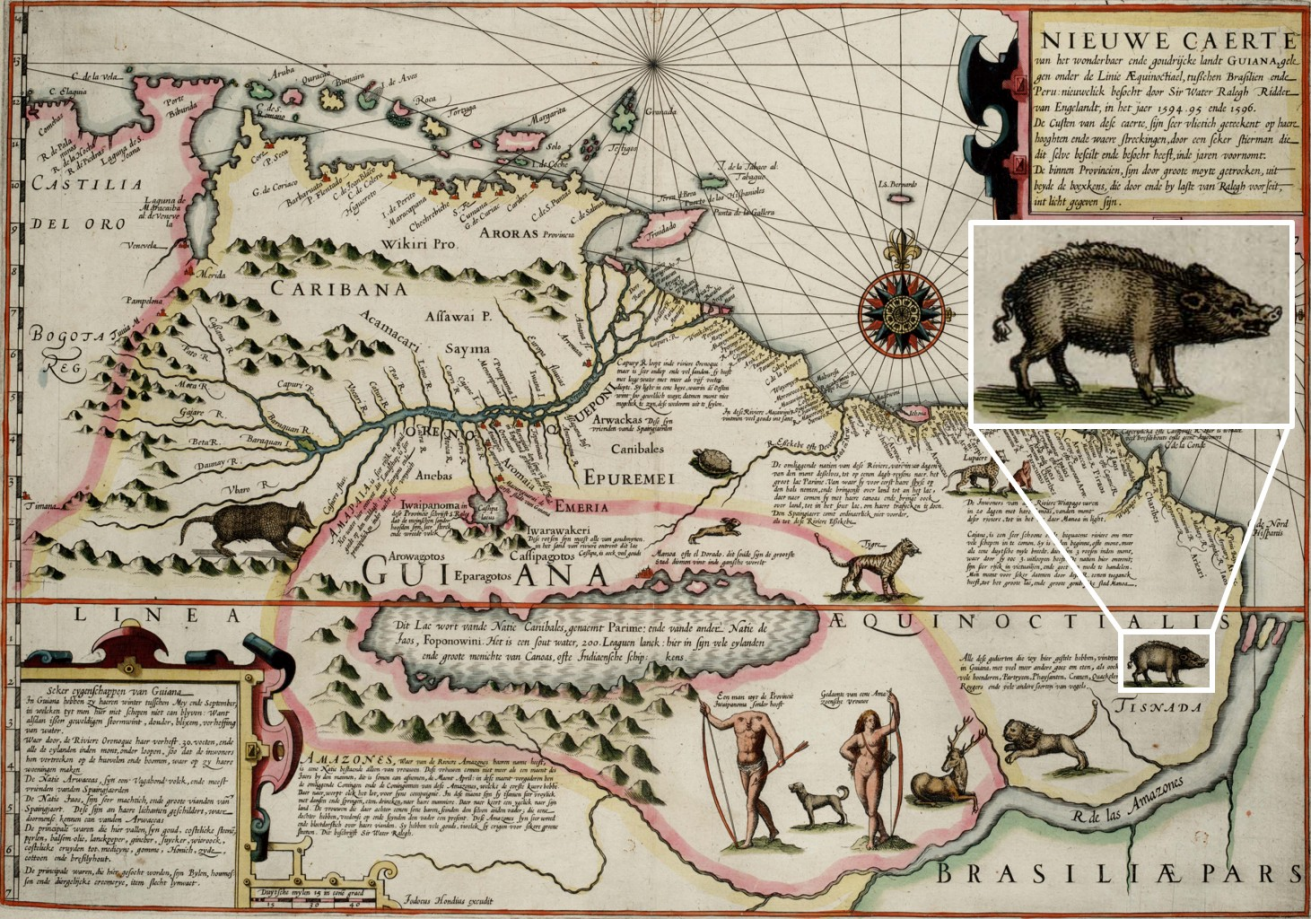
Map of the Guianas, Nieuwe caerte van het Wonderbaer ende Goudrjcke Landt Guiana. Drafted by Jodocus Hondius ca. 1598. Inset: native peccary. Map credit: public domain, https://commons.wikimedia.org/wiki/File:1599_Guyana_Hondius.jpg, image cropped from original with inset added.

Beyond Schomburgk’s (p. 259 in [17]) previously mentioned speculation that the so-called “hogges” encountered by the early English were an introduction of native peccary, there is ancillary evidence to support this proposed explanation. The *Nieuwe caerte van het Wonderbaer ende Goudrjcke Landt Guiana*, drafted by Flemish mapmaker Jocodus Hondius in the late 1590s, depicts colonial interests in the Guianas region (Fig 6). The map is embellished with mythological elements, such as headless Blemmyes and the city of Manoa (location of El Dorado), but also depicts various native fauna as a means of cataloging the plenitude of the New World. Among these animals is a peccary, accompanied by the inscription, “All the animals that we mention here, one can find in Guiana, with many others good to eat, as well as many chickens, partridges, pheasants, cranes, quails, herons, and many other sorts of birds.” (translation from Dutch by M. Kappers, personal communication 2018). The map demonstrates European awareness of a native type of “pig” suitable as game and suggests a potential source for the animals introduced to Barbados. A Spanish/Portuguese introduction would be in keeping with the European practice of island game stocking, in this case substituting wild New World fauna for Old World livestock.

### Explanation 3: A colonial English introduction

A final possibility is that the colonial English themselves introduced peccary from South America or Trinidad to Barbados. In several places in his volume, Ligon (p. 58, 104–105 in [10]) bemoans the lack of game animals for hunting on Barbados. This was a problem throughout the West Indian islands, which are depauperate of large mammals, and one reason for European introduction of Old World fauna. Peccaries are generally considered good game. In the 20^th^ century, *P*. *tajacu* was introduced for hunting to Cuba, the U.S. state of Arizona, Scotland, and Gabon [38]. *T*. *pecari* was introduced to Cuba in 1930 but is not found in the wild today [75, 81]. To the best of our knowledge, no such event is documented historically for Barbados. However, English settlers are known to have introduced fallow deer (*Dama dama*) to Barbuda for hunting [82].

An English-initiated peccary introduction could have occurred before or after Ligon’s time, but the terminal 18^th^ century date for the Chancery Lane specimen places a *terminus ante quem* on this event. The opportunity for translocation occurred early in Barbados history. In 1627, after depositing settlers on the island, the *William and John*, commanded by Captain Henry Powell sailed for a Dutch settlement in Guyana to obtain various native crops suitable for growing on Barbados, such as manioc (*Manihot esculenta*), maize (*Zea mays*) and tobacco (*Nicotiana* sp.). These were brought to the island along with 40 mainland Amerindians to instruct the settlers in local plant cultivation (pp. 5–6 in [11]). Peccaries may have been part of this economic package imported from the continent. Alternatively, peccaries could have been introduced later to offset declining pig populations. In his journal recording a 1631 voyage to Barbados upon the *Alexander*, Sir Henry Colt relates that the early English settlers had nearly hunted the “wilde hoggs” into extermination within just a few years of settlement, and usually “killed 1500 a week, a waste to[o] great to be continued.” (p. 92 in [83]). This number is undoubtedly an exaggeration. It implies the slaughter of ca. 300,000 animals in the three to four years following initial English settlement and an original pig population density of untenable proportions. Certainly, Ligon does not mention any such mass killing in his extensive discussion of pigs.

### Synthesis

The balance of evidence indicates a ca. 16^th^ century Spanish/Portuguese introduction of peccary is the most likely of the historical scenarios reviewed here. It is consistent with the known presence of a swine-like animal on Barbados upon English arrival and supported by the fact that the Spanish/Portuguese had both access to peccaries on the mainland and opportunity to introduce them to the island. Based on the radiocarbon date for the Chancery Lane specimen, this would suggest that peccaries survived on the island for at least ~30 and possibly up to ~175 years following initial English settlement, a period during which they would likely have coexisted with introduced *S*. *scrofa*. Wild pigs and peccaries were often confused until the 19^th^ century and today are still regularly mistaken for each other in places where they co-occur [9, 84]. We contend the early colonial English simply did not register a conceptual and/or linguistic distinction between the peccaries they observed on Barbados and feral pigs, leaving us with no clear textual record for the former’s presence.

It follows that the “pigs” encountered by the English upon first arrival and documented by Ligon in a *True & Exact History of the Island of Barbados* were in fact peccaries. Notably, the “swine” illustrated on Ligon’s maps take two physical forms: a smooth-skinned type and a hairy type. Caution is warranted in making species identifications from artistic renderings, but these could represent, respectively, captive domestic pigs and peccaries. Ligon may have illustrated these dimorphic forms under the impression they represented husbanded and feral hogs. Interestingly, Colt (p. 67 in [84]) specifically noted in his journal the presence of “wild hoggs [and] English hoggs”, providing support for the existence of two distinct types on Barbados.

Since peccary no longer occurs on Barbados, its introduction should be considered an ephemeral or failed event. The brevity of peccary presence (possibly only one or two centuries) does not preclude environmental consequences, however, although these remain undetermined. While *S*. *scrofa*, *P*. *tajacu* and *T*. *pecari* occupy similar ecological niches [84], because they exhibit different life history and behavioral traits, their ecological impacts are expected to differ. Correct identification of the introduced mammal encountered by English settlers on Barbados therefore carries ecological significance. *S*. *scrofa* is notoriously bioinvasive on islands. Across the Caribbean and Polynesia, it has been implicated in the failed forest regeneration, degradation of endemic species habitat, and plant and animal extinctions due to its destructive rooting habits and predation on small, ground-dwelling vertebrates and invertebrates [85, 86]. Peccaries are primarily seed predators. Information on their ecological effects in novel habitats is limited [38]. In their native Neotropics they affect forest community composition and biodiversity by influencing plant recruitment, distribution, and species richness [87]. Where modern pigs and peccaries overlap in their ranges, they coexist through niche partitioning involving diet separation and temporal shifts in foraging [88, 89]. Potential impacts from *P*. *tajacu* or *T*. *pecari* on Barbados’ environment may have been more profound in the period before English arrival where, in the absence of natural predators and human hunting, peccary herds could have become quite large. Following English settlement, deforestation for agriculture and the introduction of commensal fauna and Old World livestock would have readily outstripped any environmental effects wrought by peccary. Notably, our findings indicate impacts specific to invasive pigs did not occur on Barbados until after English settlement.

## Conclusion

Results from ^87^Sr/^86^Sr analysis and AMS radiocarbon dating of the Chancery Lane peccary specimen indicate a local animal on Barbados between AD 1645–1670 or 1780–1800 and establish a new record for non-native species introduction to the West Indies. Based on historical and archaeological data, this peccary’s presence is most likely attributable to a 16^th^ century Spanish or Portuguese translocation originating from the Guianas, Brazil, or Trinidad. Accordingly, our results revise history: the herds of abundant wild hogs encountered by English settlers upon arrival to Barbados and described by Richard Ligon in a *True & Exact History* were in fact descendants of these previously introduced peccaries. Further investigation is required to determine the species, *T*. *pecari* or *P*. *tajacu*, involved and how long it persisted on the island, data which may help to clarify the ecological impact of this ultimately failed introduction. The systematic documentation of anthropogenic faunal introductions in the pre-modern era is a critical component to understanding island historical ecology and the legacy of bioinvasion over deep time.

Equally important, this research advances mapping of the West Indian Sr isoscape and associated human and animal mobility studies through the addition of bioavailable Sr data for Barbados. It also clarifies our understanding of the process at play during a historically significant moment–the economic, political and social transformation of the Old World and the New following the Columbian encounter. As a historical document, Ligon’s map is viewed as a testament to the rapid landscape change catalyzed by the English in the first three decades of settlement and an exemplary of “ecological imperialism” [90] effected through the introduction of Old World species, land clearance and apportionment, and a slave-based agro-economy. Our results challenge some of the primacy of that narrative, suggesting Europeans selectively chose to engage with and make use of New World fauna in historically contingent ways that have previously evaded our scrutiny.
